# High glucose promotes regulatory T cell differentiation

**DOI:** 10.1371/journal.pone.0280916

**Published:** 2023-02-02

**Authors:** Elise Pitmon, Eileen Victoria Meehan, Elham Ahmadi, Adam J. Adler, Kepeng Wang

**Affiliations:** Department of Immunology, School of Medicine, University of Connecticut Health Center, Farmington, CT, United States of America; Universite Paris-Saclay, FRANCE

## Abstract

The consumption of processed foods and sugary sodas in Western diets correlates with an increased incidence of obesity, metabolic syndromes such as type 2 diabetes, cardiovascular diseases, and autoimmune diseases including inflammatory bowel disease and rheumatoid arthritis. All these diseases have an inflammatory component, of which T lymphocytes can play a critical role in driving. Much has been learned regarding the importance of sugar, particularly glucose, in fueling effector versus regulatory T cells that can promote or dampen inflammation, respectively. In particular, glucose and its metabolic breakdown products via glycolysis are essential for effector T cell differentiation and function, while fatty acid-fueled oxidative phosphorylation supports homeostasis and function of regulatory T cells. Nevertheless, a critical knowledge gap, given the prevalence of diabetes in Western societies, is the impact of elevated glucose concentrations on the balance between effector versus regulatory T cells. To begin addressing this, we cultured naïve CD4^+^ T cells with different concentrations of glucose, and examined their differentiation into effector versus regulatory lineages. Surprisingly, high glucose promoted regulatory T cell differentiation and inhibited Th1 effector differentiation. This skewing towards the regulatory lineage occurred via an indirect mechanism that depends on lactate produced by activated glycolytic T cells. Addition of lactate to the T cell differentiation process promotes the differentiation of Treg cells, and activates Akt/mTOR signaling cascade. Hence, our findings suggest the existence of a novel feedback mechanism in which lactate produced by activated, differentiating T cells skews their lineage commitment towards the regulatory fate.

## Introduction

The consumption of sugar has been rising in Western countries. In parallel, the incidences of obesity, metabolic syndrome, and cardiovascular diseases have also increased [1], as has the prevalence of autoimmune diseases such as inflammatory bowel disease (IBD), multiple sclerosis (MS), and rheumatoid arthritis (RA) [2, 3]. Indeed, the trend towards increasing autoimmune disease incidence can be seen in countries with a high pace of socio-economic improvement and westernization [2, 3]. While genetic factors predispose individuals towards the development of many inflammatory autoimmune diseases, they cannot explain the rapid increase in these Western diet-associated diseases [2]. In fact, most of these inflammatory autoimmune diseases have a relatively low concordance rate between monozygotic twins [4], suggesting that environmental factors play a dominant role. One environmental factor, sugar consumption, has been linked to increased risk for obesity and other chronic diseases [5], so it is important to understand how elevated glucose levels impact physiological mechanisms.

Given that the immune system, and in particular, T lymphocytes, play a central etiological role in inflammatory and autoimmune diseases [6, 7], there is interest in understanding how sugar regulates T cell function. For instance, glycolytic breakdown of glucose is essential for the rapid proliferation of naïve T cells following antigenic stimulation that leads to their clonal expansion and differentiation into effector T cells [8–11]. Differentiated effector T cells subsequently target infectious agents or tumors, or inappropriately cause pathologic inflammation or target self-tissues to instigate autoimmunity [12]. Nevertheless, naïve T cells can differentiate into a variety of functional lineages that have different metabolic substrate requirements [7, 9–11]. Thus, in contrast to effector T cells whose critical functions rely primarily on glucose and glycolytic metabolism, the homeostasis of regulatory T cells (Tregs) that function to control inflammation and autoimmunity mainly utilize fatty acid-driven oxidative phosphorylation [13]. Importantly, Tregs are metabolically flexible and can sustain energy production with metabolites such as lactate that can feed the tricarboxylic acid (TCA) cycle and drive oxidative phosphorylation [13]. Hence, the end product of glycolysis, pyruvate, can be converted to lactate by lactate dehydrogenase (LDH) and then secreted from a glycolytic cell. Neighboring cells, such as Tregs, can import this lactate and convert it back to pyruvate to be metabolized in the TCA cycle [14]. Indeed, the use of lactate by Tregs enables their accumulation within glycolytic tumors and enhances their ability to suppress anti-tumor immunity [15].

Although much has been learned regarding the basic relationships between metabolic substrate usage and energy-generating pathways in the development and function of effector versus regulatory T cell subsets, critical questions remain about how altered metabolic states associated with Western diets impact inflammatory and autoimmune diseases: specifically, how high glucose levels, the principal diagnostic criterion for diabetes, impacts the balance between effector and regulatory T cells that play diametric roles in fostering and controlling inflammation and autoimmunity, respectively. To begin addressing this question, we analyzed the effect of high glucose on T cell differentiation *in vitro*. Our results show that lactate produced under high glucose conditions functions as a driving factor in favor of naïve CD4^+^ T cells differentiating into Tregs over effector T cells.

## Methods

### Mice

C57BL/6 mice were obtained from the Jackson Laboratory and housed in a specific pathogen-free environment. Both male and female mice were used in experiments, all of which were reviewed and approved by the UConn Health IACUC.

### T cell culture

Naïve CD4^+^ T cells were isolated and purified from the spleens of C57BL/6 mice using a naïve CD4^+^ T cell isolation kit (Stemcell Technologies, Cat # 19765), and then cultured in 96-well round bottom plates with 0.5 μg/mL plate-bound anti-CD3 (Biolegend, Cat # 100340). T cells were cultured in RPMI medium (ThermoFisher Scientific, Cat #11879020) supplemented with indicated concentrations of glucose and 10% fetal bovine serum. Inducible Treg (iTreg) polarizing cultures also contained 1 μg/mL anti-CD28 (Biolegend, Cat # 102116), and either 15 ng/mL latent TGF-β (R&D Systems, Cat # 299-LT-005) or 0.5 ng/mL active TGF-β (PeproTech, Cat # 100–21). Th1 polarizing cultures contained 1 μg/mL anti-CD28, 5 μg/mL anti-IL-4 (Tonbo Biosciences, Cat # 70–7041), 3 ng/mL IL-12 (PeproTech, Cat # 210–12), and 5 ng/mL IL-2 (PeproTech, Cat # 212–12). Mixed T cell cultures contained 1 μg/mL anti-CD28, 0.5 ng/mL active or 15 ng/mL latent TGF-β, and 3 ng/mL IL-12. As indicated, some cultures included 15 mM lactate (Sigma-Aldrich, Cat # L4263) or 20 μM MCT1/2 inhibitor (Tocris Bioscience, Cat # 4960). Th17 differentiation was carried out with 1 μg/mL anti-CD28, 5 ng/mL active TGF-β, and 50 ng/mL IL-6.

### Flow cytometry

Cells were harvested at 72 hours of *in vitro* culture and stained with Live/Dead fixable exclusion dye (Tonbo Bioscience), followed by fluorochrome-conjugated antibodies in PBS with 2% fetal bovine serum and 1 mM EDTA. For intracellular cytokine staining, cells were stimulated with Cell Stimulation Cocktail (eBioscience, Cat # 00-4970-03) for 4 hours, followed by fixation and staining with a Foxp3/transcription factor staining buffer set (eBioscience, Cat # 00-5523-00). Flow cytometry analyses were performed on a BD LSRII flow cytometer. Data were analyzed with FlowJo software.

### Lactate assay

Naïve T cells were cultured under iTreg polarizing conditions for 24–48 hours before measuring lactate levels in the culture medium using a Lactate Assay Kit (Sigma-Aldrich, Cat # MAK064) and a ClarioStar plate reader. The samples were deproteinized to remove LDH and other proteins using a Deproteinizing Sample Preparation Kit-TCA (Abcam, Cat # ab204708) prior to lactate measurement. To observe absorption of lactate by fully differentiated Treg cells, Treg cells were differentiated under the stimulation of 1.25 μg/mL plate bound anti-CD3 antibody, 1 μg/mL soluble anti-CD28 antibody, 5 ng/mL active TGF-β, and 5 ng/mL IL-2 for 3 days. Differentiated Treg cells were cultured in 3 or 15 mM lactate for 24 hours, and the remaining lactate in the culture medium was measured by lactate assay.

### Western blotting

Fully differentiated Treg cells were stimulated by 3 or 15 mM lactate for 24 hours, and analyzed by standard Western blotting protocol for the following markers: Phospho-Akt T308 (Cell Signaling #13038), Phospho-Akt S473 (Cell Signaling #4060), Phospho-AS160 (Cell Signaling #8881), Phospho-p70s6k (Cell Signaling #97596), and Phospho-mTOR (Cell Signaling #5536). GAPDH (Cell Signaling #2118) was used as an internal control.

### Statistics

Multi-group comparison was analyzed by one-way ANOVA. Pairwise comparison was analyzed by *Student’s* t-test. In all statistical tests, p < 0.05 was considered significant. All experiments were repeated at least two times, and representative results are shown.

## Results

### High glucose promotes Treg differentiation

**Fig 1:**
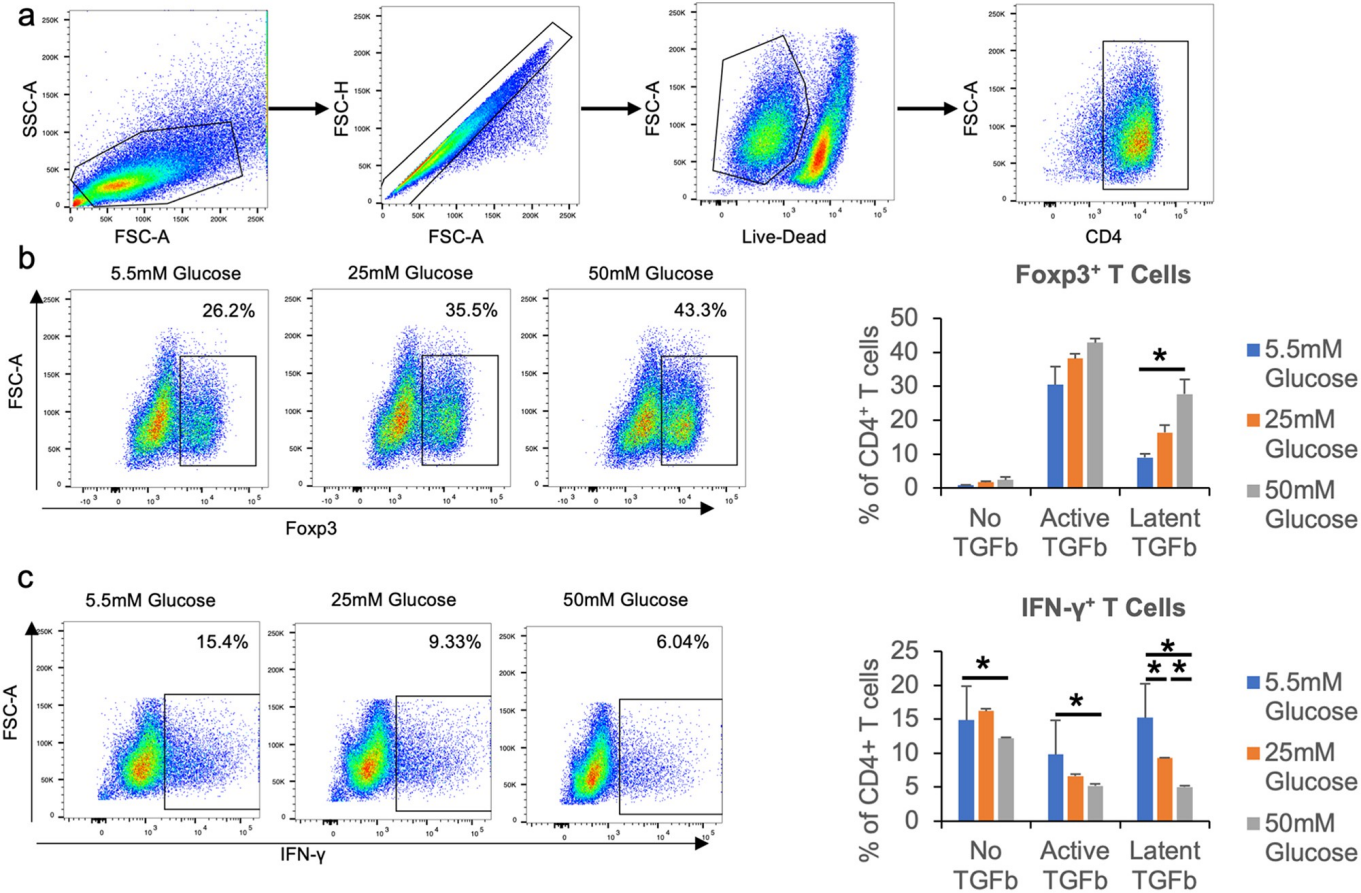
High glucose promotes iTreg differentiation. **a**–**c**, Naïve CD4^+^ T cells were isolated from C57BL/6 mice and cultured in 96-well plates with plate-bound anti-CD3 and soluble anti-CD28, and no, active, or latent TGF-β for 72 hours. Cells were then stimulated with Cell Stimulation Cocktail for 4 hours and then stained with fluorescence-conjugated antibodies and analyzed by flow cytometry. **a**, Representative flow cytometry gating for CD4^+^ T cell populations. **b**, Percentages of CD4^+^ Foxp3^+^ T cells in all CD4^+^ cells. **c**, Percentages of CD4^+^ IFN-γ^+^ T cells in all CD4^+^ cells. N = 3, and data are presented as means ± S.E.M. * p<0.05 by Student’s t test.

To test the effect of high glucose on T cell differentiation, we isolated naïve CD4^+^ T cells from C57BL/6 mice and cultured them for 72 hours in inducible Treg (iTreg) polarizing conditions (i.e., containing TGF-β) with different concentrations of glucose. Overall, the percentage of naïve CD4^+^ T cells that gained expression of the Treg master transcription factor Foxp3 increased in parallel to the glucose concentration, suggesting that high glucose can promote the differentiation of iTregs (Fig 1a and 1b). This effect was more pronounced and statistically significant when latent compared to active TGF-β was added to the cultures (Fig 1b), consistent with a previous study where latent TGF-β was required to observe an effect of glucose on T cell differentiation [16]. Reciprocally, the percentage of IFN-γ^+^ CD4^+^ T cells decreased as the glucose concentration increased, regardless of whether active or latent TGF-β was used (Fig 1c).

### High glucose inhibits Th1 differentiation

**Fig 2:**
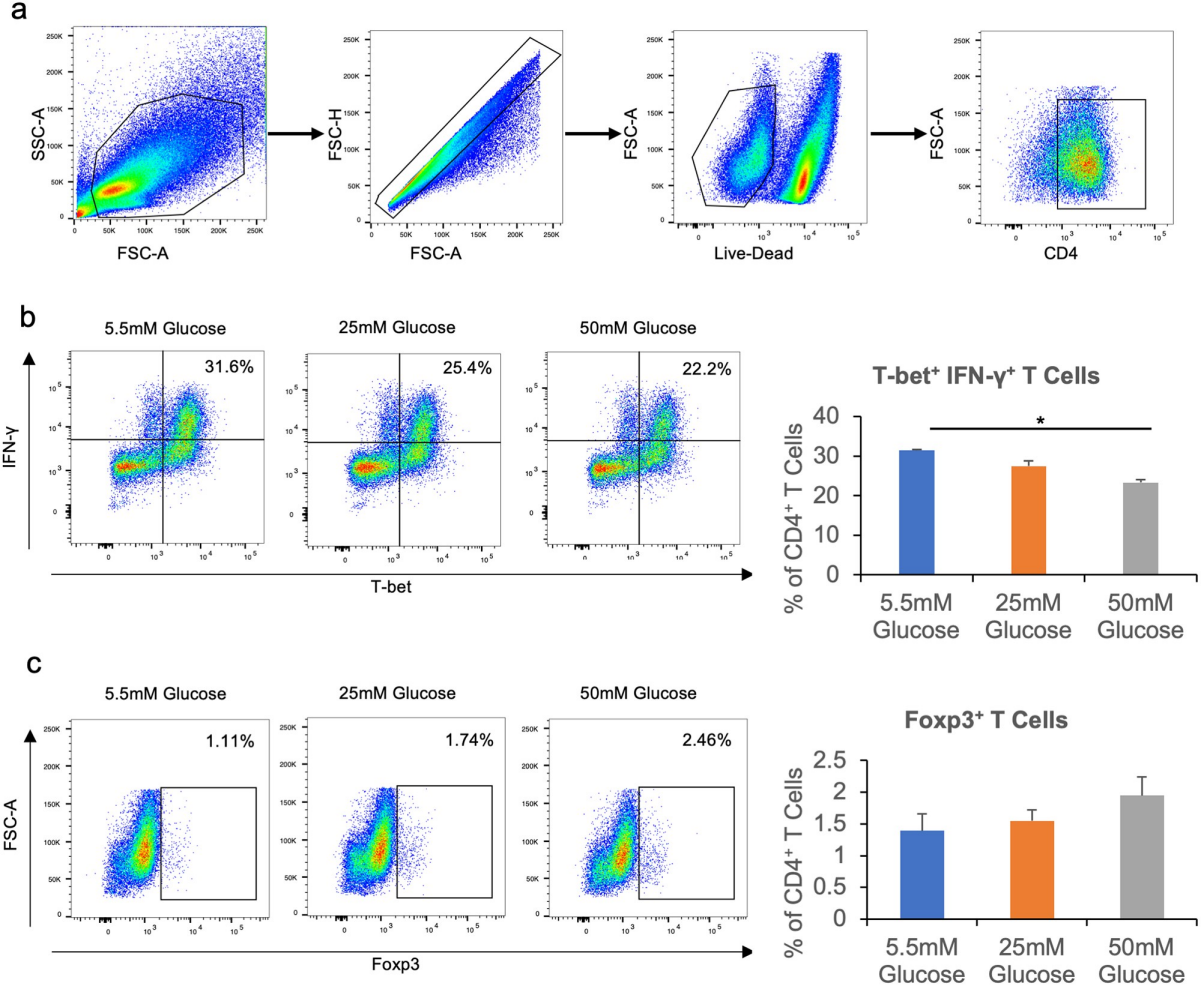
High glucose inhibits Th1 differentiation. Naïve CD4^+^ T cells were isolated from C57BL/6 mice and cultured for 72 hours in 96-well plates with plate-bound anti-CD3, and soluble anti-CD28, anti-IL-4, IL-12, and IL-2. Cells were then stimulated with Cell Stimulation Cocktail for 4 hours before staining with fluorescence-conjugated antibodies and analyzed with flow cytometry. **a**, Representative flow cytometry gating for CD4^+^ T cell populations. **b**, Percentages of CD4^+^ T-bet^+^ IFN-γ^+^ T cells in all CD4^+^ cells. **c**, Percentages of CD4^+^ Foxp3^+^ T cells in all CD4^+^ cells. N = 3, and data are presented means ± S.E.M. * p<0.05 by Student’s t test.

Th1 is the default *in vitro* differentiation pathway for naïve C57BL/6 CD4^+^ T cells, even when lineage-skewing cytokines are not added to the culture. Thus, even in our iTreg polarizing culture with the addition of TGF-β, many naïve T cells still gained the capacity to express the prototypical Th1 cytokine IFN-γ (Fig 1c). Given that high glucose in the media inhibited naïve T cells from expressing IFN-γ in the presence of TGF-β (Fig 1c), we next asked whether high glucose could also inhibit Th1 differentiation under optimal Th1-skewing conditions. Hence, naïve CD4^+^ T cells were cultured in the presence of IL-12, and without TGF-β. High glucose in the culture medium inhibited the differentiation of naïve T cells into Th1 cells that express both IFN-γ as well as the Th1 lineage-specifying transcription factor T-bet (Fig 2b). Further, a small fraction of the CD4^+^ T cells differentiated into Tregs in these IL-12-containing cultures, and their percentage increased slightly, albeit not statistically significantly, as the glucose concentration increased (Fig 2c).

### High glucose favors Treg differentiation under mixed cytokine conditions

**Fig 3:**
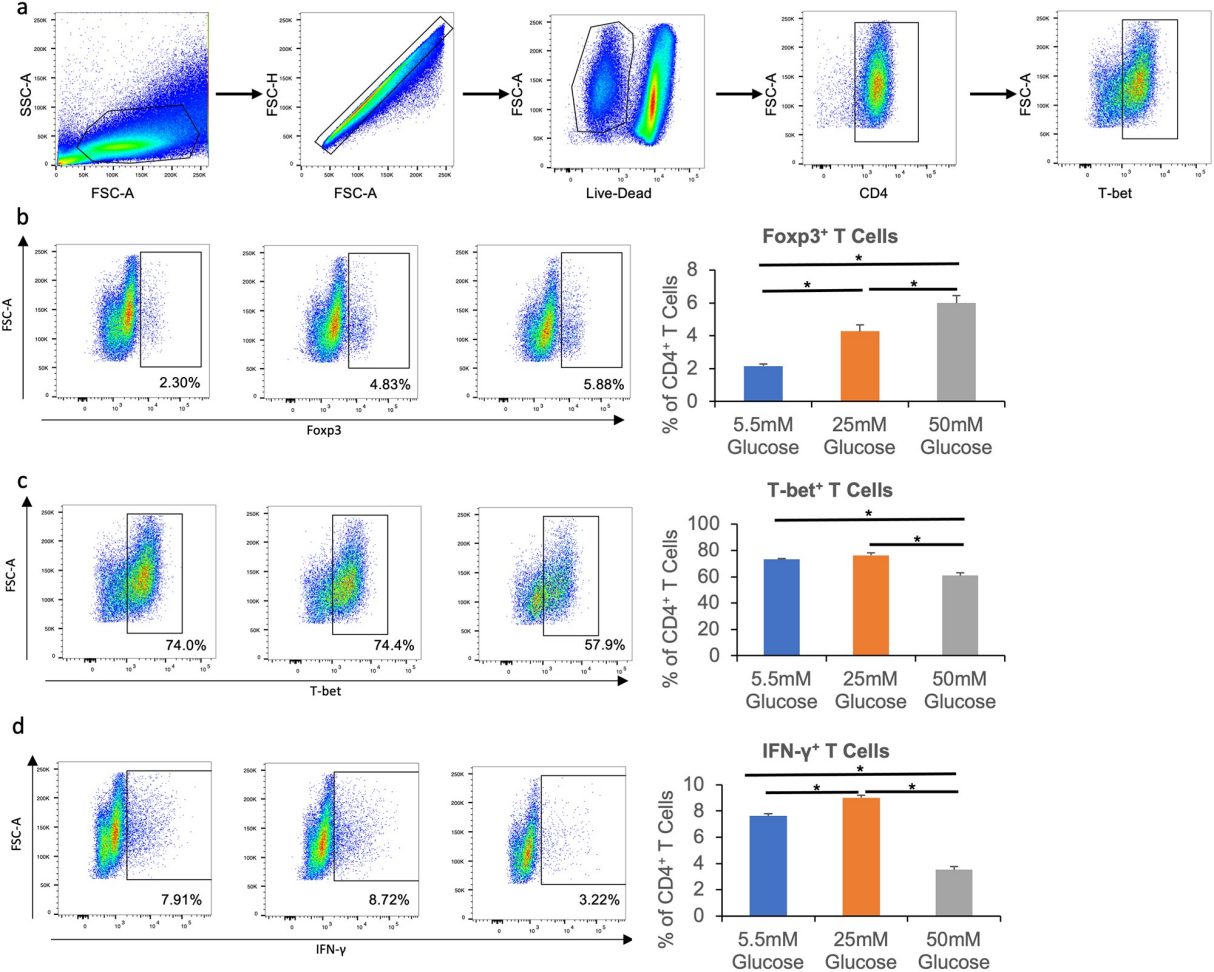
High glucose promotes Treg and inhibits Th1 differentiation under mixed cytokine conditions. Naïve CD4^+^ T cells were isolated from C57BL/6 mice and cultured in 96-well plates with plate-bound anti-CD3, and soluble anti-CD28, IL-12, and latent TGF-β for 72 hours. Cells were then stimulated with Cell Stimulation Cocktail for 4 hours before staining with fluorescence-conjugated antibodies and analyzed with flow cytometry. **a**, Representative flow cytometry gating for T cell populations. **b**, Percentages of CD4^+^ Foxp3^+^ T cells in all CD4^+^ cells. **c**, Percentages of CD4^+^ T-bet^+^ T cells in all CD4^+^ cells. **d**, Percentages of CD4^+^ IFN-γ^+^ T cells in all CD4^+^ cells. N = 3, and data are presented means ± S.E.M. * p<0.05 by Student’s t test.

In contrast to the Treg and Th1 differentiation conditions used in the preceding experiments where naïve CD4^+^ T cells are cultured with only a single fate-determining cytokine (TGF-β or IL-12), T cell differentiation *in vivo* likely occurs under more complex conditions where multiple cytokines are present. To model whether this might alter the impact of high glucose on naive CD4^+^ T cell differentiation, we next analyzed 72-hour cultures containing IL-12 and latent TGF-β. Under this “mixed” cytokine condition, high glucose caused a significant increase in Foxp3^+^ Tregs and a corresponding decrease in both T-bet^+^ and IFN-γ^+^ T cells (Fig 3a–3d). Taken together, the results shown in Figs 1–3 support that high glucose skews naive CD4^+^ T cell differentiation towards a Treg phenotype and away from the Th1 lineage.

### High glucose promotes Treg differentiation through lactate production

**Fig 4:**
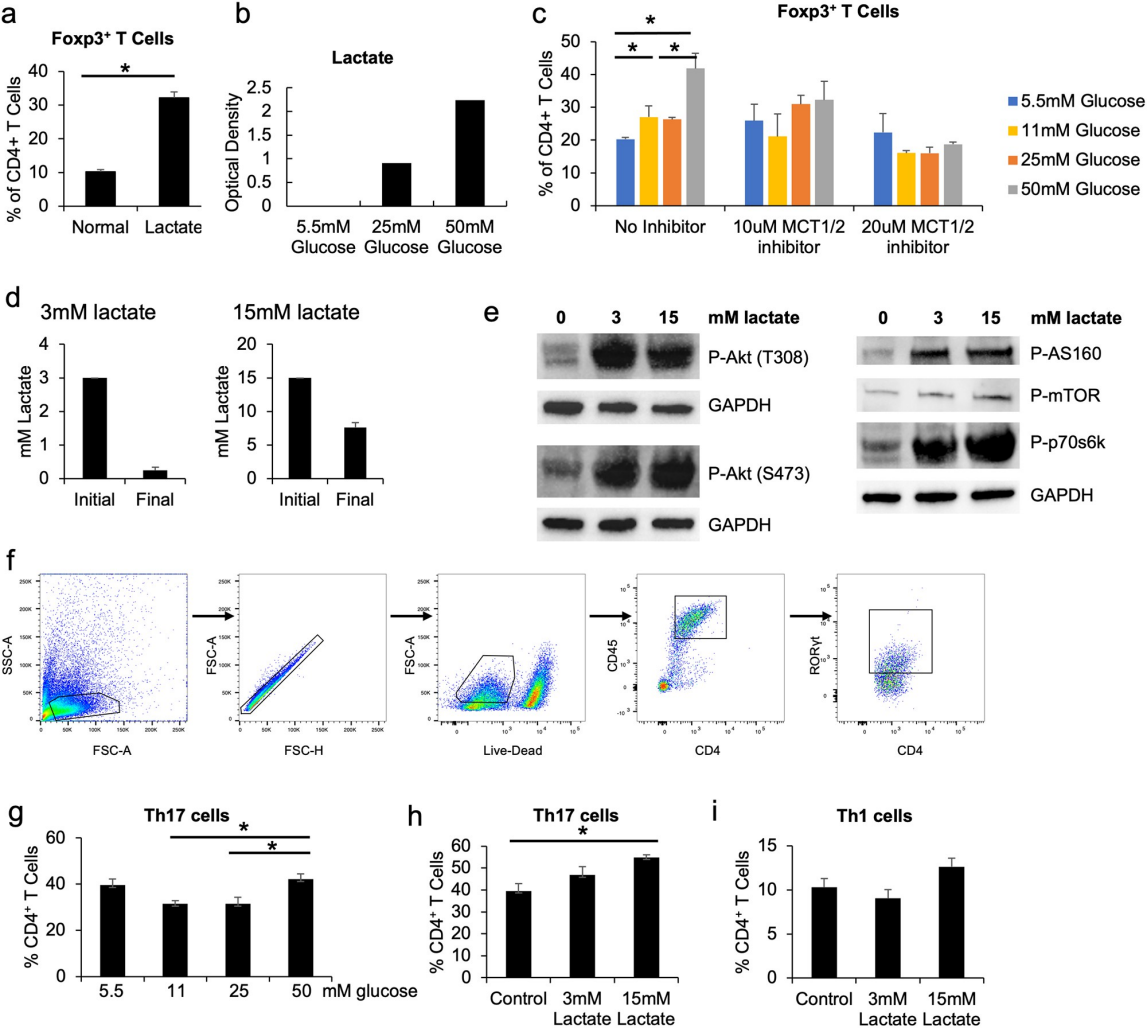
High glucose increases lactate production that promotes iTreg differentiation. **a**, Naïve CD4^+^ T cells were isolated from C57BL/6 mice and cultured in 96-well plates with plate-bound anti-CD3, soluble anti-CD28 and latent TGF-β, with or without lactate for 72 hours. Cells were then analyzed by flow cytometry for the percentage of Tregs (Foxp3^+^). **b**, Naïve CD4^+^ T cells were isolated from C57BL/6 mice and cultured in 96-well plates with plate-bound anti-CD3, soluble anti-CD28 and latent TGF-β for 24 hours. Culture supernatants were collected from each well, deproteinized, and assayed for total lactate level using a plate reader. Result represents pooled culture supernatants of 3 biological repeats. **c**, Naïve CD4^+^ T cells were cultured in 96-well plates with plate-bound anti-CD3, soluble anti-CD28, and latent TGF-β, with or without MCT1/2 inhibitor for 72 hours. Cells were then analyzed by flow cytometry for the percentage of Tregs (Foxp3^+^). N = 3, and data are presented means ± S.E.M. * p<0.05 by Student’s t test. **d**, Tregs were differentiated from naïve T cells for 3 days. Cells were then cultured in 3 (left panel) or 15 (right panel) mM lactate for 24 hours, and the concentration of the remaining lactate in the culture medium was measured by the lactate assay. **e**, Differentiated Tregs were cultured in 3 or 15 mM lactate for 24 hours, and whole cell extracts from these cells were analyzed by Western blotting for indicated markers. **f**–**h**, Naïve CD4^+^ T cells were cultured in 96-well plates with plate-bound anti-CD3, soluble anti-CD28, active TGF-β, and IL-6 for 3 days, under indicated glucose (**g**) or lactate (**h**) levels, and analyzed for Th17 differentiation. Representative gating strategy for Th17 flow cytometry are shown in **f**. **i**, Naïve CD4^+^ T cells were cultured in 96-well plates with plate-bound anti-CD3, soluble anti-CD28 and anti-IL-4, IL-2, and IL-12 for 3 days, under indicated lactate levels, and analyzed for Th1 differentiation. RPMI medium containing 5.5 mM glucose and 10% FBS was used as controls for lactate stimulation.

Next, we tested if altered metabolic byproduct production caused by high glucose is responsible for the increased Treg differentiation. In particular, lactate, an end byproduct of glycolytic breakdown of glucose, has been shown to modulate T cell function. For instance, lactate released by glycolytic cancer-associated fibroblasts causes a reduction in anti-tumoral Th1 cells while increasing Tregs [15]. To test this, we first added exogenous lactate to Treg-skewing cultures (i.e., containing latent TGF-β) with 11 mM glucose that is standard for most cell culture media. Strikingly, the addition of lactate markedly increased the percentage of differentiated Tregs (Fig 4a). Lactate may be produced by activated T cells under high glucose condition in culture. To test this, we cultured naïve T cells under antibody-mediated T cell receptor activation with increasing levels of glucose, and measured the concentration of lactate in the culture supernatants. The amount of lactate in the supernatant was not detectable in the low glucose condition, but increased as the glucose concentration was raised (Fig 4b). Subsequently, we blocked lactate import into cells using a selective inhibitor of the monocarboxylate transporter-1/2 (AR-C155858, Tocris Bioscience), which abrogated the increase in percentage of Tregs at higher glucose concentrations (Fig 4c). When cultured in medium containing 3 or 15 mM lactate, fully differentiated Tregs were found to uptake lactate from the culture medium (Fig 4d). Stimulation of Tregs with lactate also activates Akt/mTOR/p70s6k signaling cascade, and resulted in enhanced phosphorylation of AS160, which has been linked to increased glucose transportation and enhanced glycolysis (Fig 4e) [17]. TGF-β stimulates the differentiation of both Tregs and Th17 cells, where the latter also requires co-stimulation with IL-6 [18]. To test if high glucose and lactate conditions have any impact on Th17 differentiation, we cultured naïve T cells in the presence of IL-6. Differentiation of Th17 cells was enhanced by the addition of glucose or lactate (Fig 4f–4h). Lactate treatment did not significantly impact the rate of Th1 differentiation (Fig 4i). Taken together, activated CD4^+^ T cells cultured with high glucose produce increased lactate, that in turn skews their differentiation towards the Treg or related lineages.

## Discussion

Naïve and activated T cells have different metabolic substrate preferences [11, 19]. In particular, T cells switch from oxidative phosphorylation to aerobic glycolysis upon activation. Aerobic glycolysis is required for T cell effector function [19]. Uptake of glucose by Glut1 is followed by glycolytic breakdown to pyruvate with the net production of two ATP molecules. In non-proliferating and terminally differentiated T cells, pyruvate is further processed through the tricarboxylic acid (TCA) cycle to generate NADH and FADH2, which are in turn oxidized to produce 36 molecules of ATP per glucose molecule. In activated effector T cells, however, glucose-generated pyruvate is converted into lactate, even though sufficient oxygen is available for oxidative phosphorylation [20, 21]. This increased reliance on glycolysis permits an adequate supply of metabolic precursors for the biosynthesis of nucleic acids, proteins, and lipids that support clonal expansion of the effector T cells [22, 23].

In contrast to effector Th1 cells and Th17 cells, memory T cells and Tregs mainly rely on fatty acid oxidation to produce ATP [9, 24]. Thus, AMPK signaling in Tregs results in low expression of Glut1, the major glucose transporter in activated T cells, which promotes, for instance, their accumulation in a mouse model of asthma [24–26]. Here, we show that lactate produced by activated T cells promotes their commitment towards the Treg lineage. Specifically, we found that lactate production increases when activated T cells are supplied with increased concentrations of glucose, and the augmentation of Treg differentiation under high glucose conditions is mitigated by a lactate import inhibitor.

It has previously been shown that the potential for Tregs to be fueled by lactate can be coopted by glycolytic tumors as a means to establish an immunosuppressive microenvironment [15]. Our current results support that a similar process may also function within a T cell negative feedback loop that calibrates the balance between effector and regulatory T cells. Thus, to limit excessive inflammation during immune responses, Treg expansion often coincides with clonal expansion of effector T cells [27, 28]. One mechanism of these opposing T cell actions is through the secretion of IL-2 by activated effector CD4^+^ T cells that supports the expansion of neighboring CD25^+^ Tregs [29, 30]. Our current finding that lactate produced by activated glycolytic CD4^+^ T cells may thus represent a second mechanism by which the balance between effector and regulatory T cells is maintained. Further, during diabetes when glucose availability increases, excess lactate production may serve as a compensatory feedback mechanism to limit pathogenic inflammation.
